# Ascorbate Inhibits Proliferation and Promotes Myeloid Differentiation in *TP53*-Mutant Leukemia

**DOI:** 10.3389/fonc.2021.709543

**Published:** 2021-08-23

**Authors:** Carlos C. Smith-Díaz, Nicholas J. Magon, Judith L. McKenzie, Mark B. Hampton, Margreet C. M. Vissers, Andrew B. Das

**Affiliations:** ^1^Centre for Free Radical Research, Department of Pathology and Biomedical Science, University of Otago, Christchurch, New Zealand; ^2^Haematology Research Group, Christchurch Hospital and Department of Pathology and Biomedical Science, University of Otago, Christchurch, New Zealand

**Keywords:** epigenetic therapy, differentiation, ascorbate, TET2, Prima-1^Met^, APR-246, vitamin C, leukemia

## Abstract

Loss-of-function mutations in the DNA demethylase TET2 are associated with the dysregulation of hematopoietic stem cell differentiation and arise in approximately 10% of *de novo* acute myeloid leukemia (AML). *TET2* mutations coexist with other mutations in AML, including *TP53* mutations, which can indicate a particularly poor prognosis. Ascorbate can function as an epigenetic therapeutic in pathological contexts involving heterozygous *TET2* mutations by restoring *TET2* activity. How this response is affected when myeloid leukemia cells harbor mutations in both *TET2* and *TP53* is unknown. Therefore, we examined the effects of ascorbate on the SKM-1 AML cell line that has mutated *TET2* and *TP53*. Sustained treatment with ascorbate inhibited proliferation and promoted the differentiation of these cells. Furthermore, ascorbate treatment significantly increased 5-hydroxymethylcytosine, suggesting increased TET activity as the likely mechanism. We also investigated whether ascorbate affected the cytotoxicity of Prima-1^Met^, a drug that reactivates some p53 mutants and is currently in clinical trials for AML. We found that the addition of ascorbate had a minimal effect on Prima-1^Met^–induced cytotoxicity, with small increases or decreases in cytotoxicity being observed depending on the timing of treatment. Collectively, these data suggest that ascorbate could exert a beneficial anti-proliferative effect on AML cells harboring both *TET2* and *TP53* mutations whilst not interfering with targeted cytotoxic therapies such as Prima-1^Met^.

## Introduction

Acute myeloid leukemia (AML) is a hematological cancer that harbors a poor prognosis. The disease is highly heterogeneous at the genetic level, with at least 11 distinct subgroups comprising driver mutations in over 100 different genes (1). Epigenetic dysregulation is a key feature of many of these AML subgroups (2, 3). Consequently, therapeutic strategies targeting the mutations that lead to epigenetic dysregulation offer hope for novel forms of treatment.

Ascorbate is an emerging epigenetic therapeutic. This property arises from its co-factor activity for the Fe-containing 2-oxoglutarate-dependent dioxygenases, a large family that includes prolyl hydroxylases (4, 5) numerous histone demethylases (2, 6) and the ten-eleven translocation (TET) enzymes (6–8). The TET proteins are responsible for the active demethylation of DNA *via* the oxidation of 5-methylcytosine (5mC) to 5-hydroxymethylcytosine (5hmC), 5-formylcytosine (5fC), and 5-carboxylcytosine (5caC) (9–11). Ascorbate sustains and promotes TET activity, most likely by reducing Fe^3+^ to Fe^2+^ during the catalytic cycle (8). TET2 activity and cellular levels of 5hmC increase with intracellular ascorbate availability in a dose-dependent manner (12–14). Therefore, ascorbate has the potential to act as an epigenetic therapeutic *via* the stimulation of TET2 activity. This has been demonstrated *in vitro* and using mouse models (3, 15, 16). Furthermore, we have previously reported that supplementation with ascorbate resulted in clinical remission in a patient with AML harboring a *TET2* mutation (17).

TET2 is a major regulator of hematopoiesis, regulating the differentiation and self-renewal of hematopoietic stem cells (HSC) (15). This has been demonstrated in murine studies showing that *Tet2* knockout results in the expansion of the HSC population and skews the peripheral erythrocyte-to-monocyte ratio in favor of increased peripheral monocytes (18). The role of TET2 in hematopoiesis is also evident from the observation that loss-of-function mutations are frequently found in blood disorders such as AML, clonal hematopoiesis of indeterminate potential, chronic myelomonocytic leukemia and myelodysplastic syndrome (19, 20).

*TET2* mutations in AML are associated with a significant decrease in 5hmC (21), highlighting the role that this enzyme plays as an epigenetic regulator in hematopoiesis. Emerging insights also suggest that ascorbate supplementation could be beneficial in AML cases associated with decreased *TET2* activity (15, 16). However, *TET2* mutations occur in conjunction with numerous combinations of other mutations (1, 2) and we know relatively little about how other mutations might affect the ascorbate-mediated up-regulation of TET2 activity and subsequent effects on cell differentiation and survival. The epigenetic effects of ascorbate have been explored in some models of leukemia (15, 16, 22–24), but have not been considered in the context of *TP53* and *TET2* mutated AML.

*TP53* loss-of-function mutations are of clinical interest as they confer an exceedingly poor prognosis in AML. Three-year survival rates are between 0 – 15% (25) and new treatment options are desperately required. Therefore, we investigated the effects of ascorbate on growth and differentiation using SKM-1 cells - a model of leukemia where both *TET2* and *TP53* mutations co-exist (26). We also investigated the effects of ascorbate in conjunction with Prima-1^Met^ (APR-246), a compound which reactivates some p53 mutants and promotes oxidative stress in cancer cells (27–30). Prima-1^Met^ has been shown to act synergistically with azacitidine to inhibit the proliferation of a number of *TP53*-mutant cell lines, including SKM-1 (31). Given the importance of combination therapy in AML treatment, we investigated the interplay between ascorbate and Prima-1^Met^ to determine whether the antioxidant activity of ascorbate interfered with the cytotoxicity of Prima-1^Met^.

## Methods

### General Cell Culture Methods

SKM-1 cells were provided by the Dawson Lab at the Peter MacCallum Cancer Institute, Melbourne, Australia. These cells were grown in RPMI-1640 medium with penicillin, streptomycin and 10% heat-inactivated fetal bovine serum. Cells were cultured at 37°C with a humidified atmosphere of 5% CO_2_. A complete refresh of the media was carried out prior to commencing experiments by centrifugation of the cell suspension, aspiration of media and resuspension in new media.

### Validation

SKM-1 DNA was sent for analysis to CellBank Australia, confirming a 98% match between the sample DNA and a reference SKM-1 genome. PCR was used to amplify regions of DNA at the sites of the known *TET2* and *TP53* mutations in the SKM-1 cells. Primers were designed using the Benchling bioinformatics platform. Sanger sequencing at the University of Otago, Dunedin confirmed the presence of a hemizygous *TP53* mutation (c.743G>A, **Supplementary Figure 1**) and a heterozygous *TET2* mutation (c.4253_4254insTT, **Supplementary Figure 2**), consistent with other reports in the literature (26, 31). The 743G>A mutation in *TP53* is a hotspot mutation in patients, translating to a R248Q substitution in the DNA binding domain and is predicted to result in loss-of-function (1). Some studies also suggest that R248Q results in *TP53* gain-of-function attributes (32). The *TET2* mutation results in a frameshift at p.1419 with the truncation of ~600 amino acids. Given that the fundamental 2-OG-dependent dioxygenase domain of TET2 is located at amino acids 1322-2002, this frameshift most likely results in complete loss-of-function (33). Flow cytometry was used to show that the SKM-1 cells were CD11b^+^, CD117^+^, CD13^+^, CD33^+^, CD45RA^+^, CD15^+^, CD19^-^ and CD3^-^. The observed cell surface marker data is consistent with data from Deutsche Sammlung von Mikroorganismen und Zellkulturen and reports in the literature (34). In house PCR testing was used to confirm that the SKM-1 cell line was negative for mycoplasma contamination.

### Ascorbate and Phosphoascorbate Uptake

Ascorbate or phosphoascorbate was added to SKM-1 cells in a 12-well plate in a final volume of 2 ml containing 0.2 x 10^6^ cells/ml and incubated for periods up to 48 hours. At the end of the incubation period, the cells were pelleted by centrifugation at 1000 g for 5 minutes at room temperature and resuspended in PBS. An equal volume of ice cold 0.54 M perchloric acid (PCA) containing 50 µM diethylenetriaminepentaacetic acid (DTPA) was added to supernatant and cell samples. The samples were then mixed by vortex and centrifuged at 12,000 *g* for 2 minutes at 4°C to remove the protein precipitate. Extracted cell lysate and supernatant samples were stored at −20°C and the ascorbate levels were measured using HPLC with electrochemical detection as previously described (35, 36). Extracts were pre-treated with Tris(2-carboxyethyl)phosphine (TCEP) to reduce any dehydroascorbic acid (DHA) present and the DHA content was determined from the difference between the two measures (35).

### Effects of Ascorbate on Cell Proliferation, Cell Cycle and Apoptosis

SKM-1 cells were cultured for 6 days with the addition of sodium ascorbate, bovine liver catalase, and phosphoascorbate (2-phospho-L-ascorbic acid trisodium salt) where indicated. Cells were seeded at 0.2 x 10^6^ cells/ml in a volume of 1 ml in a 24-well plate. Ascorbate and phosphoascorbate were added at 0 – 500 μM and catalase at 20 μg/ml. The media was refreshed with a 1:5 dilution at days 2 and 4, with the addition of ascorbate, catalase and phosphoascorbate at the original concentration. The number of cells was recorded after 6 days with a hemocytometer and viability assessed by trypan blue dye exclusion. PI/Annexin V and PI DNA staining were used to assess cell viability/apoptosis and cell cycle status *via* flow cytometry after 6 days with 300 μM ascorbate, with data analysis using CXP Software from Beckman Coulter (Brea, CA, USA).

### Analysis of Cell Surface Markers

To assess ascorbate-mediated changes on differentiation, SKM-1 cells were grown for up to 36 days +/- 300 μM phosphoascorbate or ascorbate. Every 2-3 days the medium was refreshed by diluting 1:5 with fresh media and adding phosphoascorbate or ascorbate at the original concentration. At 7, 12/13 days, 22 days and 36 days, cell surface marker expression was analyzed using flow cytometry for CD15, CD33, CD45RA, CD117, CD13, and CD11b. The percentage change in mean fluorescence intensity (MFI) was calculated by taking the ratio of the MFI for the treated cells relative to the control, subtracting 1 and then converting to a percentage. The data was exported using CXP Software. Isotype controls were used to confirm the binding specificity of the antibodies.

### Mass Spectrometry Analysis of 5hmC Levels

SKM-1 cells were cultured with 300 µM ascorbate or phosphoascorbate in 12-well plates at 0.4 x 10^6^ cells/ml in 2 ml per well for periods up to 4 days. The medium was refreshed by diluting 1:5 in fresh media with the addition of 300 µM ascorbate or phosphoascorbate after 2 days. At the end of the incubation period, the cells were harvested and the DNA was extracted using a DNA extraction kit (DNeasy Blood and Tissue Kit Cat No. 69504, Qiagen, Hilden, Germany).

A stable isotope dilution LC-MS/MS method was used for the detection and quantification of 2’-deoxycytidine, 5-methyl-2’-deoxycytidine and 5-hydroxymethyl-2’-deoxycytidine. Isotopically labeled standards [2’-deoxycytidine (^13^C, ^15^N_2_), 5-methyl-2’-deoxycytidine (^13^C, ^15^N_2_) and 5-hydroxy-methyl-2’-deoxycytidine (d_3_)] were used to control for experimental variations such as recovery, matrix effect, and ionization. Standard calibration curves using the ratio of light to heavy isotopes were used for quantification. One µg of SKM-1 DNA was hydrolyzed using a nucleoside digestion kit M0649S (New England Biolabs, Ipswich, MA, USA) in the presence of internal standards [130 fmoles 2’-deoxycytidine (^13^C, ^15^N_2_), 5 fmoles 5-methyl-2’-deoxycytidine (^13^C, ^15^N_2_) and 0.013 fmoles 5-hydroxy-methyl-2’-deoxycytidine (d_3_)].

Standards and digested SKM-1 DNA samples were analyzed using a 6500 QTrap mass spectrometer (Sciex, Framingham, MA, USA) coupled to an Infinity 1290 LC system (Agilent, Santa Clara, CA, USA). Standards and samples were stored on the autosampler tray at 5°C. An Acclaim RSLC Polar Advantage II 120Å column (150 x 2.1 mm, Thermo Fisher Scientific Inc., Waltham, MA, USA) was used for chromatographic separation using 100% water (0.1% formic acid) as Solvent A and 100% acetonitrile (0.1% formic acid) as Solvent B. A flow rate of 0.2 mL/minute was used. The column temperature was set to 40°C. The analytes were eluted during the initial isocratic phase with 100% Solvent A over 3.5 minutes. The column was then flushed with 5% Solvent A and 95% Solvent B for 2.5 minutes, and then re-equilibrated at initial conditions for 5 minutes. Data were analyzed using Analyst 1.7.1 (Sciex, Framingham, MA, USA). All species were quantified by fragmenting the singly-charged parent ion [M+H]^+^, monitoring the fragment ion resulting from the loss of the deoxyribose sugar in positive-ion mode (**Table 1**), and measuring the area under the curve of the resulting peak (Fit: Linear, Weighting: None, Regression Parameter: Area). The concentration of deoxycytidine, 5mC and 5hmC in each sample was calculated by relating the peak area ratio of the light to the heavy isotope to standard calibration curves, and then converted to a percentage of the total cytidine species. The assay was validated by measuring the relative composition of cytidine species in frontal cortex and liver tissues (Dunkin Hartley guinea pigs) for comparison. For guinea pig tissue samples, one µg of DNA was hydrolyzed in the presence of 340 fmoles 2’-deoxycytidine (^13^C, ^15^N_2_), 10 fmoles 5-methyl-2’-deoxycytidine (^13^C, ^15^N_2_) and 1.3 fmoles 5-hydroxy-methyl-2’-deoxycytidine (d_3_)).

**Table 1:** The m/z values for the singly-charged parent and fragment ions and the optimised parameters that were used to quantify each analyte in LC-MS/MS experiments.

Analyte	Parent (m/z)	Fragment (m/z)	DP	EP	CE	CXP
2’-Deoxycytidine (H^+^)	228.10	112.05	45	7	15	16
2’-Deoxycytidine (^13^C, ^15^N_2_) (H^+^)	231.10	115.05	45	7	15	16
5-Methyl-2’-deoxycytidine (H^+^)	242.11	126.07	45	7	13	16
5-Methyl-2’-deoxycytidine (^13^C, ^15^N_2_) (H^+^)	245.11	129.06	45	7	13	16
5-Hydroxy-methyl-2’-deoxycytidine (H^+^)	258.11	142.06	50	7	11	16
5-Hydroxy-methyl-2’-deoxycytidine (d_3_) (H^+^)	261.13	145.08	50	7	11	16

DP, declustering potential; EP, entrance potential; CE, collision energy; CXP, cell exit potential.

### Effects of Prima-1^Met^ and Ascorbate

SKM-1 cells were cultivated in a 24-well plate in 1 ml at a starting concentration of 0.2 x 10^6^ cells/ml. Cells were pre-treated with either 300 μM ascorbate or phosphoascorbate for 4 hours before the administration of Prima-1^Met^. Cell viability was assessed at 24 hours by resuspending the cells in PBS with 20 µg/mL propidium iodide (PI) and analyzed by flow cytometry.

SKM-1 cells were also grown with or without 300 μM ascorbate or phosphoascorbate for 1 week. The cells were seeded at 0.4 x 10^6^ cells/ml in 2 ml in a 12-well plate. The media was refreshed with a 1:5 dilution every 2-3 days, with the addition of ascorbate or phosphoascorbate at the original concentration. The cells were then centrifuged at 1000 *g* for 5 minutes and resuspended in fresh media at a cellular concentration of 0.2 x 10^6^ cells/ml. The cells were left for 1 hour in the incubator before transfer into a 24-well plate and treatment with Prima-1^Met^. Cell viability was assessed at 24 hours by resuspending the cells in PBS with 20 µg/mL PI and analyzed by flow cytometry.

### Statistical Analysis

The results in this paper are expressed as means ± the standard error of the mean unless indicated otherwise. Prism 9 software (GraphPad, La Jolla, CA, USA) was used for statistical analysis. The ascorbate-mediated inhibition of cell growth and increases in 5hmC levels were analyzed by one-way ANOVAs with Dunnett’s multiple comparison post-hoc test. Paired t-tests (2-tailed) were used for comparing the changes in the expression of SKM-1 cell surface proteins and cell viability after growth in ascorbate/phosphoascorbate replete media. Two-way ANOVAs with Tukey’s post-hoc tests were used to analyze the combined cytotoxic effect of Prima-1^Met^ and ascorbate/phosphoascorbate, and differences in intracellular ascorbate concentrations. For all tests the statistical significance was set at p < 0.05.

### Materials

Nucleoside standards were obtained from Toronto Research Chemicals (Toronto, Ontario, Canada). FITC and PE conjugated fluorescent antibodies were obtained from Biolegend (San Diego, CA, USA), CD15 (Cat. 301903), CD33 (Cat. 366619), CD45RA (Cat. 304105), CD117 (Cat. 313203), CD13 (Cat. 301703), CD11b (Cat. 301305), CD3 (Cat. 100203), CD9 (Cat. 312105). The drug Prima-1^Met^ (CAS No: 5291-32-7) was obtained from MedChemExpress, Monmouth Junction, NJ, USA.

## Results

### Ascorbate and Phosphoascorbate Uptake Dynamics

**Figure 1:**
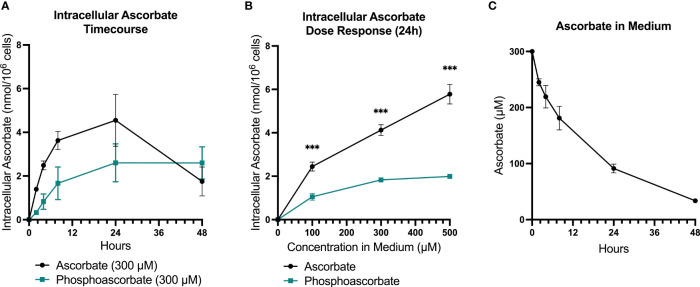
SKM-1 Ascorbate and phosphoascorbate uptake dynamics. The ascorbate content of SKM-1 cells following the addition of varying concentrations of ascorbate or phosphoascorbate to RPMI medium for up to 48 hours. SKM-1 cells were seeded at 0.2 x 10^6^ cells/ml in a volume of 2 ml in 12-well plates and were harvested at the times shown. **(A)** The intracellular concentration of ascorbate (n = 6) or phosphoascorbate (n = 3) was measured at different time intervals following the addition of 300 μM ascorbate or phosphoascorbate to the medium. **(B)** Intracellular ascorbate levels at 24 hours following the addition of 0 – 500 μM ascorbate or phosphoascorbate to the medium (n = 3) ***p < 0.001, representing a significant difference between the intracellular concentration after ascorbate or phosphoascorbate treatment. **(C)** The ascorbate concentration in the media (containing SKM-1 cells) at different time intervals following the addition of 300 μM ascorbate (n = 3).

RPMI cell culture medium does not contain ascorbate, and therefore SKM-1 cells maintained under standard culture conditions lack ascorbate. Following the supplementation of ascorbate to the medium, intracellular ascorbate levels increased in a dose-dependent manner, with levels peaking at 8-24 hours and decreasing again by 48 hours (**Figures 1A, B**). When 300 μM ascorbate was added to the medium, the intracellular levels peaked around 4 nmoles/10^6^ cells at 8-24 hours after ascorbate administration, with significant variation between batches of cells. By 48 hours, very little ascorbate remained in the medium, although intracellular levels remained around 2 nmol/10^6^ cells (**Figures 1A, C**). Most of the cellular ascorbate was present as reduced ascorbate: DHA was estimated to be 5.26 ± 2.4% of the total ascorbate (SEM, n=15), determined by measuring ascorbate with and without pre-treatment of the cell extracts with TCEP.

High ascorbate concentrations in cell culture media result in the generation of extracellular H_2_O_2_ *via* the reduction of traces of free iron present in solution. This effect can contribute to ascorbate-mediated cytotoxicity in cell culture (37). For this reason, we included controls that mitigated any H_2_O_2_-induced toxicity by: (i) adding catalase to the medium, or (ii) using the redox-stable molecule phosphoascorbate. Phosphoascorbate is an ascorbate analogue that does not undergo oxidation to DHA and thus circumvents the generation of H_2_O_2_ in cell culture media (22, 38, 39). When phosphoascorbate was added to the medium instead of ascorbate, SKM-1 intracellular ascorbate levels also increased, but at a slower rate. The maximum intracellular ascorbate concentration after phosphoascorbate administration to the medium was measured at around 2-2.5 nmoles/10^6^ cells (**Figures 1A, B**).

### Ascorbate Inhibits SKM-1 Cell Proliferation

**Figure 2:**
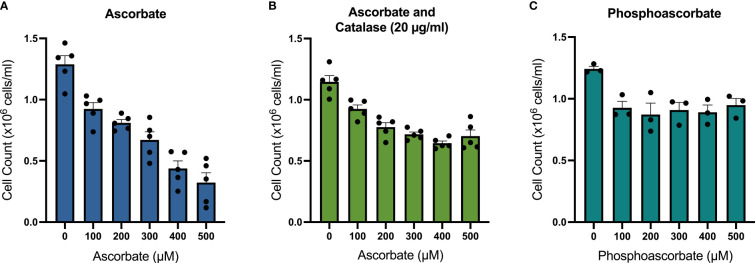
Effect of ascorbate and phosphoascorbate on SKM-1 cell growth. **(A–C)** SKM-1 cell numbers 6 days following treatment with ascorbate (n = 5), ascorbate and catalase (n = 5) or phosphoascorbate (n = 3). Each data point represents the mean of two cells counts recorded with a hemocytometer from two separate wells with the same treatment. The cells were all seeded at a starting concentration of 0.2 x 10^6^ cells/ml, with 1:5 dilutions after 2 days and 4 days in cell culture media with re-administration of ascorbate, phosphoascorbate and catalase at the original concentration. The setup of this experiment means that if the cells had not grown at all, then the total cell count after 6 days would be around 8 x 10^3^ cells/ml. All treatments resulted in a statistically significant inhibition of cell growth. 100 μM ascorbate +/- catalase p < 0.01; 200 - 500 μM ascorbate +/- catalase p < 0.0001; 100, 500 μM phosphoascorbate p < 0.05; 200 - 400 μM phosphoascorbate p < 0.01.

**Figure 3:**
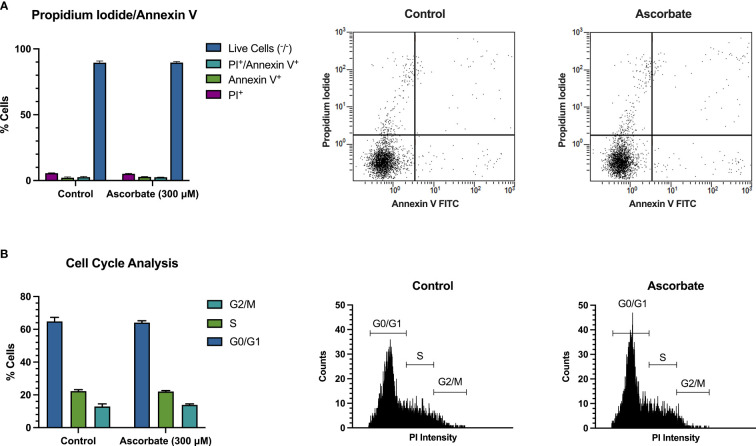
300 μM ascorbate does not promote cell death or affect the cell cycle. SKM-1 cells were grown for 6 days in media with 300 μM ascorbate. **(A)** Staining with annexin V/FITC and propidium iodide shows that 300 μM ascorbate does not promote cell death or apoptosis (n = 3). **(B)** Cell cycle analysis shows no effect on the cell cycle (n = 3). Representative traces are shown.

SKM-1 cell proliferation was significantly decreased when the cells were maintained in ascorbate-replete media for 6 days. With 500 μM ascorbate in the medium, we noted a 75% inhibition of cell growth (**Figure 2A**). When 20 μg/ml catalase was added to the medium to control for the possible contribution of H_2_O_2_-induced cytotoxicity, cell growth was inhibited in a concentration-dependent manner up to 300 μM ascorbate, with a maximum inhibition of 44% (**Figure 2B**). When phosphoascorbate was added to the medium instead of ascorbate, cell proliferation was decreased at 100 μM, with no further decrease at higher concentrations (**Figure 2C**). Exposure to 300 μM ascorbate for 6 days had no effect on cell viability, apoptosis or the cell cycle (**Figures 3A, B**) and no loss in viability was observed after 28 hours growth in media with 300 μM ascorbate or phosphoascorbate. Furthermore, staining with trypan blue and observation by microscopy indicated that the cells maintained a high degree of viability (between 0-1 trypan blue positive cells per 200 cells). Collectively these data indicate that ascorbate inhibits SKM-1 cell growth without promoting cell death at culture media concentrations between 100 to 300 μM. They also indicate that the inhibition of cell growth is not dependent on H_2_O_2_ at these concentrations.

### Ascorbate Modulates Expression of SKM-1 Cell Surface Proteins

**Figure 4:**
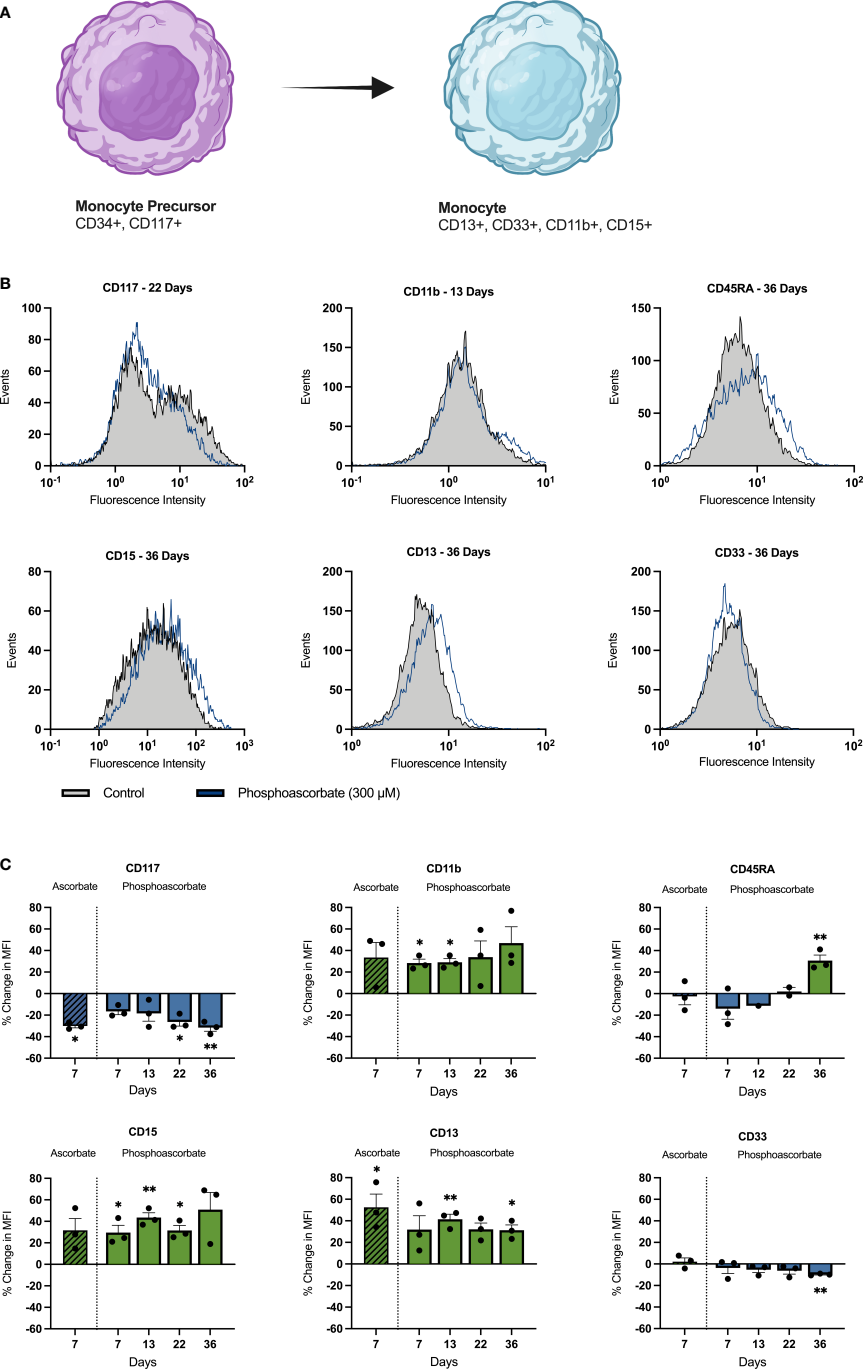
Ascorbate-induced changes in cell surface antigen expression. **(A)** Changes in cell surface markers associated with monocytic differentiation. Common granulocyte/monocyte precursor cells express immature cell antigens such as CD34 and CD117 which persist up until the monocyte precursor/monoblast differentiation stage. Once these immature cells mature into promonocytes and mature monocytes, CD117 and CD34 expression is lost and the cells begin to express CD15, CD33, CD13 and CD11b along with other characteristic cell surface markers (40). These changes are mirrored by SKM-1 cells following growth in ascorbate-replete media with notable increases in CD15, CD13, CD11b and a decrease in CD177. **(B)** Representative histograms showing shifts in the fluorescence intensity signal for each cell surface marker after treatment with phosphoascorbate, relative to the control. **(C)** Relative changes in cell surface antigen expression following treatment with phosphoascorbate and ascorbate. Measurements are n = 3 except for CD45RA expression, which was measured at n = 1 (at 12 days) and n = 2 (at 22 days). For all other measurements the 13-day timepoint includes one measurement taken at 12 days and two measurements at 13 days. For CD117, when two distinct subpopulations were present, the change in MFI was calculated by gating to select the CD117^+^ subpopulation and calculating the MFI change for this subpopulation. Statistically significant changes are marked as: *p < 0.05, **p < 0.01. Parts of this figure were made using BioRender.com.

Growth in cell culture media with 300 μM phosphoascorbate promoted changes in cell surface markers consistent with monocytic differentiation (**Figure 4A**). Significant changes in several cell surface proteins were observed, with increases in CD13 (13, 36 days), CD15 (7, 13, 22 days), CD11b (7, 13 days), CD45RA (36 days), and decreases in CD117 (22, 36 days) and CD33 (36 days) (**Figures 4B, C**). Similar changes were also achieved with 300 μM ascorbate at 7 days (**Figure 4C**). The direction of these changes is consistent with monocytic cell differentiation and maturation.

### Ascorbate Increases 5hmC Levels

**Figure 5:**
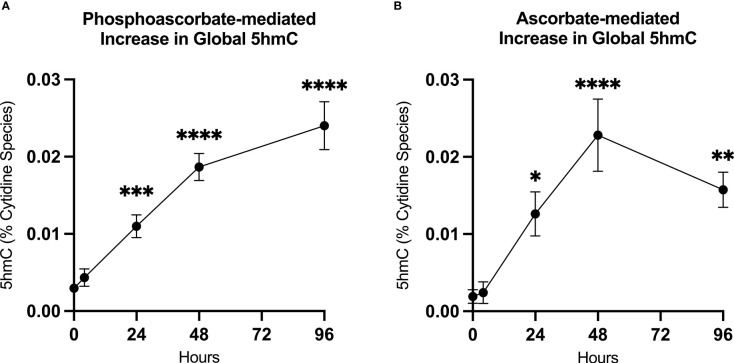
Ascorbate induced changes in 5hmC levels. **(A, B)** Increase in 5hmC in SKM-1 cells following growth in media with 300 μM phosphoascorbate or ascorbate. p-values are displayed for statistically significant increases relative to the control. ****p < 0.0001, ***p < 0.001, **p < 0.01, *p < 0.05. All time points represent at least n = 3 except for ascorbate 4 hours which is n = 2. Treatment with ascorbate or phosphoascorbate had no apparent effect on global methylation.

We utilized mass-spectrometry to measure the relative abundance of deoxycytidine, 5mC and 5hmC in untreated cells at 95.5% ± 0.4, 4.5% ± 0.4, 0.003% ± 0.001 of the total cytidine species respectively (average ± SD, n=9). We then observed that the addition of either 300 μM ascorbate or phosphoascorbate to the cell culture media resulted in increased global 5hmC. This effect was time-dependent; culturing the SKM-1 cells with ascorbate or phosphoascorbate for up to 2 days resulted in progressively higher levels of 5hmC (**Figures 5A, B**). After 4 days with phosphoascorbate, 5hmC levels had increased 8-fold relative to untreated cells. Treatment with ascorbate or phosphoascorbate had no observable effect on global methylation in either direction. We observed that SKM-1 5hmC levels, even after growth in media with ascorbate, were still lower than levels measured in tissue samples (**Table 2**).

**Table 2:** The measured 5mC and 5hmC levels in SKM-1 cells grown with and without ascorbate/phosphoascorbate, and reference tissue samples (average ± SD).

Sample	5mC (% total cytidine species)	5hmC (% total cytidine species)
SKM-1 (n=9)	4.5% ± 0.4	0.003% ± 0.001
SKM-1 (48h phosphoascorbate, n=4)	4.2% ± 0.7	0.019% ± 0.004
SKM-1 (48h ascorbate, n=4)	4.2% ± 0.6	0.023% ± 0.009
Liver (guinea pig, n=4)	3.7% ± 0.7	0.076 ± 0.022
Frontal Cortex (guinea pig, n=4)	4.0% ± 0.3	0.75% ± 0.11

### Effects of Ascorbate on Prima-1^Met^ Induced Cytotoxicity

**Figure 6:**
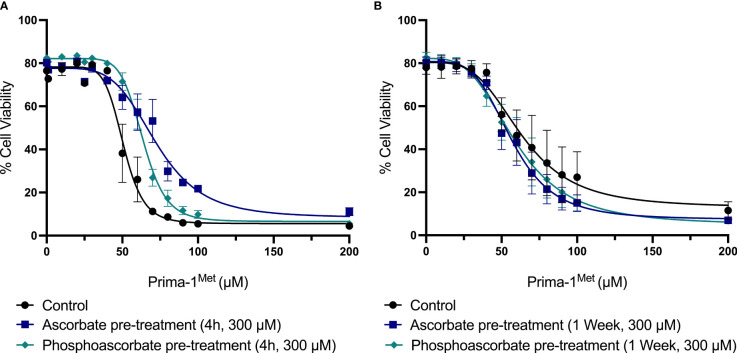
The effect of ascorbate on Prima-1^Met^ cytotoxicity. **(A)** SKM-1 cells were grown for 4 hours +/- ascorbate or phosphoascorbate (300 μM). The cells were all seeded at a starting concentration of 0.2 x 10^6^ cells/ml at a depth of 1 ml in a 24 well plate. After 4 hours, Prima-1^Met^ was added and the percentage cell viability was calculated after 24 hours using PI and flow cytometry (n = 3). For reference, clinical studies show that Prima-1^Met^ is tolerated at plasma concentrations up to 250 μM with only relatively minor side effects (41). Pre-treatment with either ascorbate or phosphoascorbate significantly decreased the cytotoxicity of Prima-1^Met^ (p < 0.0001). **(B)** SKM-1 cells were grown for 1 week +/- ascorbate or phosphoascorbate (300 μM). The media was refreshed every 2/3 days with a 1:5 dilution. The cells were then resuspended in fresh media, diluted to a concentration of 0.2 x 10^6^ cells/ml and left for 1 hour before the addition of Prima-1^Met^. The percentage cell viability was then measured 24 hours later (n = 4). Pre-treatment with either ascorbate or phosphoascorbate for 1 week significantly increased the cytotoxicity of Prima-1^Met^ (ascorbate p = 0.0046, phosphoascorbate p = 0.0397).

The addition of 300 μM ascorbate or phosphoascorbate to the culture medium 4 hours prior to the administration of Prima-1^Met^ increased the LC_50_ after 24 hours (**Figure 6A**). In cells pre-incubated with ascorbate or phosphoascorbate for 4 hours the LC_50_ for Prima-1^Met^ was 72 μM and 64 μM respectively whereas the control was 51 μM. However, the reverse effect was observed when cells were grown in ascorbate-replete media for 1 week to induce changes in cell surface differentiation markers, before treatment with Prima-1^Met^ (**Figure 6B**). After 1 week pre-treatment with ascorbate the LC_50_ was 63 μM for the control, 57 μM for ascorbate pre-treated cells and 59 μM for phosphoascorbate pre-treated cells. These differences were small but statistically significant. It is important to note that the LC_50_ differs for the control between the two sets of experiments (51 μM vs 63 μM) which may be due to differences in experimental setup. However, a comparison has been made for each treatment relative to its own control. Overall, ascorbate pre-treatment only exhibited a small influence on Prima-1^Met^ induced cytotoxicity.

## Discussion

Dysregulated epigenetics is a known driver of AML (2, 3). Mutations in proteins such as TET2, DNMT, IDH1/2, and WT1 are common in AML, and all affect epigenetic processes (2, 17). Recently, scientists have begun to consider the possibility of employing ascorbate to target epigenetic dysregulation in AML, by harnessing its ability to stimulate TET2 (15, 16). Agathocleous et al. showed that ascorbate availability could affect HSC proliferation and differentiation, demonstrating an increase in HSCs relative to body mass and decreased levels of HSC 5hmC in ascorbate-deficient *Gulo* ^−/−^ mice (16). Upon administering ascorbate to a *FLT3^ITD^ TET2^+/-^ Gulo* ^−/−^ murine model of leukemia, overall survival was prolonged and the progression of the disease was suppressed (16). Cimmino et al. also observed that knocking down TET2 with RNAi led to aberrations in the self-renewal capacity of HSC, an effect that was reversed once TET2 activity was restored. The restoration of TET2 activity promoted cell death, myeloid differentiation and DNA demethylation. Moreover, the administration of ascorbate pharmacologically mimicked TET2 restoration (15). These findings suggest that intracellular ascorbate levels are linked to cell differentiation and 5hmC levels.

However, few studies have investigated how additional mutations might affect the TET2-dependent benefit of ascorbate treatment in AML. In a model of AML characterized by *TET2* and *TP53* loss-of-function, we have now shown that ascorbate inhibits proliferation, increases 5hmC and drives cellular differentiation. Ascorbate also had minimal effect on the efficacy of Prima-1^Met^, a novel cytotoxic reagent currently in clinical trial for AML with *TP53* mutations (31, 42). This is significant given the adverse prognosis associated with *TP53* mutations and the fact that the TP53/complex karyotype subgroup of AML has been identified as comprising 13% of cases (1).

Ascorbate is not generally added to culture media and cultured cells are usually ascorbate deficient (43–45). We observed that supplementing the cell culture medium with ascorbate resulted in rapid uptake into cells and was associated with a significant decrease in proliferation at 6 days. Extended incubation with ascorbate also induced differentiation towards a more mature cell phenotype over a 36-day period. Differentiation is fundamentally an epigenetic process and can be initiated by a variety of cell intrinsic and extrinsic cues. Given that SKM-1 cells carry a heterozygous loss-of-function mutation in the epigenetic eraser *TET2*, we hypothesized that the restoration of TET2 activity was a likely mechanism for these phenotypic changes. Consistent with this hypothesis, we found that treatment with ascorbate increased 5hmC - a proxy for TET activity. These findings highlight the potential for epigenetic therapy in this subtype of leukemia.

In untreated SKM-1 cells, 5hmC levels were very low compared with levels reported in non-cancerous human tissues (46) and the levels we measured in guinea pig frontal cortex and liver samples. This phenomenon has been previously observed in human cell lines and cancers, both of which can have very low levels of 5hmC relative to their tissue of origin (46, 47). For example, in one study, 5hmC levels in healthy colorectal tissue were found to be ~10-fold higher compared with levels in colorectal cancer tissue (47). Moreover, it has been observed that cells may undergo a loss in global 5hmC as they adapt to cell culture conditions (46). The effect may be partially attributed to reduced TET activity in a cell culture environment lacking ascorbate, although Nestor et al. also observed significantly reduced TET expression in human cell lines (46). We observed that supplementing the cell culture media with ascorbate or phosphoascorbate caused a significant increase in 5hmC within 24 hours and an ~8-fold increase in 5hmC after 48 hours growth with ascorbate.

The increased expression of SKM-1 cell surface proteins CD13, CD15, CD11b and decreased expression of CD117 after growth in ascorbate-replete media is consistent with monocytic differentiation (40). CD11b expression increases in the U937 promonocytic cell line following treatment with 12-O-tetradecanoylphorbol-13-acetate to induce cellular differentiation (48). CD117 expression, in contrast, is associated with an immature cell phenotype (40). CD117 is involved in stem cell differentiation and is generally lost as progenitor cells differentiate into mature blood cells, with the exception of dendritic and mast cells (49–51). The significance of the observed decrease in CD33 and increase in CD45RA is less clear. CD33 expression increases in the context of monocytic maturation, however in other cellular contexts, such as neutrophil maturation, a slight decrease in CD33 expression is observed during cellular maturation (40). Overall, the observed changes in cell surface protein expression following growth in ascorbate-replete media are consistent with other findings in the literature. For example, Cimmino et al. reported that reactivating TET2 caused a similar decrease in CD117 expression along with increased CD34 and CD11b expression (15). When the cell surface marker data is considered collectively, the general trend of the changes in surface protein expression points to cellular differentiation and mirrors the changes observed during monocytic differentiation.

The oxidation of ascorbate in solution, and particularly in cell culture media that contain traces of ferrous iron, is known to generate H_2_O_2_ (3, 52). This can reach cytotoxic levels when ascorbate concentrations approach 1 mM (52–54). We employed two separate strategies to avoid this artefact: we added catalase to the medium to scavenge any extracellular H_2_O_2_ and used phosphoascorbate, a redox inert substitute for ascorbate. Phosphoascorbate is hydrolyzed by membrane-bound phosphatases prior to uptake *via* the sodium-vitamin C transporters and accumulates in the cells as reduced ascorbate, with dephosphorylation being the rate limiting step for cellular uptake (55). It is likely that the slower rate of intracellular ascorbate accumulation when phosphoascorbate was used reflects the requirement for phosphoascorbate to be hydrolyzed by membrane-bound phosphatases. Both ascorbate and phosphoascorbate resulted in similar intracellular levels after 48 hours.

Adding 300 μM ascorbate or phosphoascorbate to the medium resulted in intracellular ascorbate levels similar to those measured *in vivo*: Agathocleous et al. measured intracellular ascorbate levels at around 2.5 fmoles/cell (or 2.5 nmoles/10^6^ cells) in hematopoietic stem cells from Gulo^+/+^ mice (16), which is comparable to the 2-4 nmoles/10^6^ cells measured in the SKM-1 cells. This data gives us confidence that the ascorbate-mediated effects on hydroxymethylation, differentiation, and growth inhibition in the SKM-1 cells were achieved at intracellular ascorbate concentrations comparable to those seen *in vivo* and were not driven by H_2_O_2_ generated in the cell culture media. Overall, we found that phosphoascorbate was very useful for our cell culture experiments. We recommend that future cell culture studies with ascorbate use phosphoascorbate to limit the pro-oxidant effects of ascorbate in cell culture.

Loss-of-function in p53 can be caused by mutations that result in a truncated protein, disrupted protein folding or a mutation in a DNA contact residue (56). In the case of a protein folding mutation it is sometimes possible to pharmacologically stabilize the mutant protein and restore a functional conformation (57). This exciting prospect has prompted the search for small molecule compounds that can promote functional p53 folding, such as Prima-1^Met^ (41). Prima-1^Met^ is a pro-drug that is converted to methylene quinuclidinone (MQ), which can act as a Michael acceptor, rendering it susceptible to nucleophilic attack from the sulfhydryl moiety of cysteine residues. MQ has been shown to bind Cys124 and Cys277 in p53 to promote the reactivation of R175H and R273H p53 mutants by stabilizing the protein and shifting the equilibrium in favor of an active conformation (58, 59). In addition to p53-dependent effects, MQ targets the cellular redox balance *via* binding to glutathione and thioredoxin reductase, causing the depletion of glutathione and the inhibition of thioredoxin reductase (30, 41).

Interestingly, ascorbate supplementation had divergent effects on Prima-1^Met^ cytotoxicity depending on the order and timing of treatment. The preincubation of SKM-1 cells with ascorbate for 1 week to promote cellular differentiation caused a small, but statistically significant increase in the cytotoxicity of Prima-1^Met^. In contrast, the reverse effect was observed when cells were preincubated with ascorbate for only 4 hours, with a decrease in the cytotoxicity of Prima-1^Met^. The protective effect of ascorbate after 4 hours might be rationalized on the basis that dosing with ascorbate just before Prima-1^Met^ allows SKM-1 cells to better survive the generation of reactive oxygen species. On the other hand, the enhanced cytotoxic effect of ascorbate after 1 week could be a result of ascorbate induced cellular differentiation rendering the cells more susceptible to Prima-1^Met^. However, in all cases the effect size was small and thus the biological significance of these effects remains unclear. Clinical trial data will be required to determine the utility of ascorbate as an adjunct therapy in this scenario.

In addition to *TET2* mutations, there are potentially other scenarios where TP53/complex karyotype AML patients could benefit from ascorbate supplementation. For example, decreased TET2 activity can also arise as a result of mutations in *IDH1, IDH2* or *WT1* (2) and mutations in this pathway collectively occur in around 30-50% of AML (1, 60, 61). Ascorbate-depletion also decreases TET2 activity, with similar effects to loss-of-function mutations (2, 15, 16), and low plasma ascorbate levels are common in AML patients (62, 63). Importantly, in a recent clinical trial, oral ascorbate supplementation increased 5hmC levels in myeloid cancer patients on azacitidine treatment (64).

Overall, we have found that ascorbate has the potential to function as an epigenetic therapeutic in a *TP53*-mutant leukemia model. Moreover, our data does not contraindicate the use of both ascorbate and Prima-1^Met^, as ascorbate had a negligible effect on cytotoxicity caused by Prima-1^Met^.

## Data Availability Statement

The raw data supporting the conclusions of this article will be made available by the authors, without undue reservation.

## Author Contributions

AD, MV, and MH conceived the work that led to this manuscript. CS-D, NM, JM, MH, MV, and AD designed the experiments and interpreted the data. CS-D, NM, and AD, carried out the experiments. CS-D, MH, MV, and AD wrote the paper with contributions from all authors. All authors contributed to the article and approved the submitted version.

## Funding

This study was supported by funding from the Canterbury Medical Research Fund, the Bone Marrow Cancer Research Trust, and a NZSO Roche Translational Cancer Research Fellowship to AD.

## Conflict of Interest

The authors declare that the research was conducted in the absence of any commercial or financial relationships that could be construed as a potential conflict of interest.

## Publisher’s Note

All claims expressed in this article are solely those of the authors and do not necessarily represent those of their affiliated organizations, or those of the publisher, the editors and the reviewers. Any product that may be evaluated in this article, or claim that may be made by its manufacturer, is not guaranteed or endorsed by the publisher.
