# Supplementary material for: Ascorbate Inhibits Proliferation and Promotes Myeloid Differentiation in TP53-Mutant Leukemia
Source: Front Oncol. 2021 Aug 23;11:709543. doi: 10.3389/fonc.2021.709543 (PMC8419345; doi:10.3389/fonc.2021.709543)
Supplement: Supplementary file 1 [file DataSheet_1.pdf]

Supplementary Data

TET2 Wild Type Allele Sequence	TET2 Mutated Allele Sequence
5'GGTATGCACTCTCACTAGAGAAGACAATCGAGAATTTGGA<br>GGAAAACCTGAGGATGAGCAGCTTCACGTTCTGCCTTTATAC<br>AAAGTCTCTGACGTGGATGAGTTTGGGAGTGTGGAAGCTCA<br>GGAGGAGAAAAAACGGAGTGGTGCCATTCAGGTACTGAGTT<br>CTTTTCGGCGAAAAGTC 3'	*5'GGTATGCACTCTCACTAGAGAAGACAATCGAGAATTTG<br>GAGGAAAACCTGAGGATGAGCAGCTTCACGTTCTTTGCCT<br>TTATACAAAGTCTCTGACGTGGATGAGTTTGGGAGTGTGG<br>AAGCTCAGGAGGAGAAAAAACGGAGTGGTGCCATTCAGG<br>TACTGAGTTCTTTTCGGCGAAAAGTC 3'*

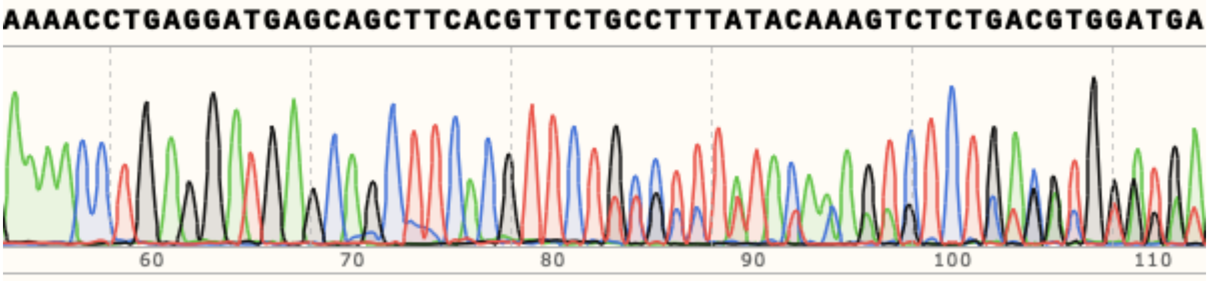

Supplementary Figure 1: TET2 Sequence Data
Extracts of the base pair sequences obtained for the two TET2 alleles in the SKM-1 cells. The mutated allele contains a heterozygous TT insertion (highlighted in yellow). These sequences were derived from the electropherogram data. The TET2 forward primer electropherogram is included above. This data illustrates the heterozygous TT insertion at base pair position 84.

TP53 Wild Type Allele Sequence (Reference Genome)	TP53 Mutated Allele Sequence (SKM-1 Cells)
5'TCTTGGGCCTGTGTTATCTCCTAGGTTGGCTCTGACTGTAC<br>CACCATCCACTACAACTACATGTGTAACAGTTCCTGCATGGG<br>CGGCATGAACCGGAGGCCCATCCTCACCATCATCACACTGG<br>AAGACTCCAGGTCAGGAGCCACTTGCCACCCTGCACACTGG<br>CCTGCTGTGCCCCAGCCTCTGCTT 3'	***5'**TCTTGGGCCTGTGTTATCTCCTAGGTTGGCTCTGACTGT<br>ACCACCATCCACTACAACTACATGTGTAACAGTTCCTGCA<br>TGGGCGGCATGAACCAGAGGCCCATCCTCACCATCATCA<br>CACTGGAAGACTCCAGGTCAGGAGCCACTTGCCACCCTG<br>CACACTGGCCTGCTGTGCCCCAGCCTCTGCTT **3'***

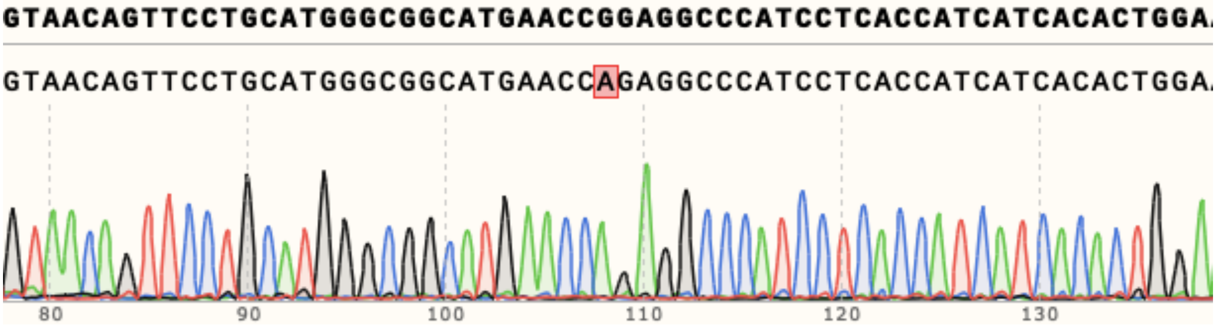

Supplementary Figure 2: TP53 Sequence Data
Extract of the base pair sequence for the fragment of the SKM-1 DNA corresponding to the site of the *TP53* mutation along with the electropherogram for the *TP53* forward primer. The electropherogram shows the homozygous substitution of an A in the place of a G at base pair position 108.
